# Effect of Ergocalciferol on β-Cell Function in New-Onset Type 1 Diabetes: A Secondary Analysis of a Randomized Clinical Trial

**DOI:** 10.1001/jamanetworkopen.2024.1155

**Published:** 2024-03-05

**Authors:** Benjamin Udoka Nwosu, Sadichchha Parajuli, Rohit B. Sharma, Austin F. Lee

**Affiliations:** 1 Northwell Health, Division of Endocrinology, Department of Pediatrics, Cohen Children’s Medical Center, New Hyde Park, New York; 2 Division of Endocrinology, Department of Pediatrics, University of Massachusetts Medical School, Worcester; 3 Monroe Family Medicine Program, Louisiana State University Hospital, Shreveport; 4 Division of Endocrinology, Diabetes, and Metabolism, Department of Medicine, Weill Cornell Medicine, New York, New York; 5 Department of Population and Quantitative Health Sciences, University of Massachusetts Chan Medical School, Worcester

## Abstract

This secondary analysis of a randomized clinical trial evaluates the effectiveness of ergocalciferol vs placebo in youths with newly diagnosed type 1 diabetes.

## Introduction

Approximately 30% to 50% residual β-cell function may remain at the time of type 1 diabetes (T1D) diagnosis, and this may persist for months or years.^1,2^ A prolonged partial remission (PR) phase of T1D leads to improved glycemic control and decreased long-term complications.^1^ We previously reported^1^ that ergocalciferol significantly decreased circulating tumor necrosis factor (TNF)-α and temporal trends in both hemoglobin A_1c_ (HbA_1c_) and insulin dose–adjusted A_1c_ (IDAA_1c_), a marker of PR, compared with placebo. Here, we report the effect size of high-dose ergocalciferol (50 000 IU/wk for 2 months, then biweekly for 10 months) vs placebo on β-cell function, denoted by the ratio of fasting proinsulin to C-peptide (PI:C) and the percent change from baseline in the area under the curve (%∆AUC) of C-peptide.

## Methods

We conducted a post hoc secondary analysis of a single-center, double-blind, placebo-controlled, parallel-group randomized clinical trial of ergocalciferol vs placebo in youths (aged 10-21 years) with newly diagnosed T1D. The trial was conducted at the University of Massachusetts Medical Center (UMMC) in Worcester from October 19, 2017, to April 12, 2021 (ClinicalTrials.gov identifier: NCT03046927). The methodology was published previously.^1^ The UMMC Institutional Review Board approved the study protocol (Supplement 1) with modifications (Supplement 2).^3^ We obtained written informed consent from adults and parents and assent from youths (aged <18 years). The study followed the CONSORT reporting guideline.

Exclusion and inclusion criteria were reported previously^1^ and included fasting C-peptide (>0.1 nmol/L [0.3 ng/mL]) or stimulated C-peptide (≥0.2 nmol/L [≥0.6 ng/mL]) and a positive diabetes-associated autoantibody profile. Participants entered a run-in phase of 1 to 2 months, maintained a treat-to-target insulin regimen, and were subsequently randomized to ergocalciferol or placebo. Participants completed mixed-meal tolerance tests to estimate C-peptide, fasting glucose, and proinsulin (normal range, 3.6-22 pmol/L) at 0, 3, 6, 9, and 12 months (Table).

**Table.** Fasting Proinsulin, Fasting C-Peptide, and Corresponding PI:Cs During the Randomized Clinical Trial

Group	Mean (SD)		
	Proinsulin, pmol/L	C-peptide, pmol/L	PI:C
Ergocalciferol, mo			
0	9.56 (16.91)	265.2 (271.6)	0.028 (0.015)
3	5.13 (5.25)	195.7 (133.2)	0.025 (0.011)
6	5.25 (6.60)	152.7 (95.7)	0.052 (0.099)
9	4.68 (4.11)	140.0 (78.4)	0.040 (0.053)
12	2.75 (2.11)	114.5 (69.4)	0.026 (0.013)
Placebo, mo			
0	10.08 (19.01)	225.9 (131.7)	0.039 (0.071)
3	6.31 (5.26)	215.0 (126.9)	0.026 (0.016)
6	8.13 (10.99)	238.7 (187.4)	0.031 (0.017)
9	5.14 (5.01)	146.1 (78.0)	0.029 (0.012)
12	6.82 (9.51)	166.2 (155.6)	0.039 (0.022)

Abbreviation: PI:C, proinsulin to C-peptide ratio.

The sample size and power calculation were reported previously.^1^ Statistical analysis was based on the intent-to-treat principle. We compared differences in fasting PI:C trends between groups using a repeated-measures generalized linear model with normal distribution, logarithmic link function, and unstructured correlations.

We calculated %∆AUC C-peptide using the trapezoidal method^4,5^ and used a random intercept model adjusted for sex, age, and race to characterize mean %∆AUC C-peptide from 0 to 12 months. Statistical analyses were performed using SAS, version 9.4 (SAS Institute Inc). Box plots were generated using Prism, version 10 (GraphPad Inc). All tests were 2 sided; *P* < .05 was considered significant. Data analysis was performed from October 5 to November 15, 2023.

## Results

Of 48 youths with T1D eligible for the 12-month trial, 36 (24 males [66.7%], 12 females [33.3%]) were randomized to ergocalciferol or placebo (eFigure in Supplement 3). Their mean (SD) age was 13.5 (2.8) years; 2 were Asian (5.6%), 2 were Black (5.6%), 27 (75.0%) were White, and 5 (13.8%) did not report their race. Ergocalciferol significantly decreased fasting PI:C vs placebo (mean [SE], −0.0009 [0.0008] vs 0.0011 [0.0003]; *P* = .01; Figure, A and B) for the monthly overall difference in trends. The mean (SD) decrease in %∆AUC C-peptide was similar for both groups in the first 3 months (−10.9 [6.3] vs −8.2 [7.0]; *P* = .99) but subsequently decreased more slowly with ergocalciferol vs placebo (−28.4 [6.2]; *P* < .001 vs −41.5 [5.9]; *P* < .001), with a significant reduction in monthly overall temporal trends (mean [SE], −2.8% [0.7] vs −4.7 % [0.6]; *P* = .03; Figure, C).

**Figure.**
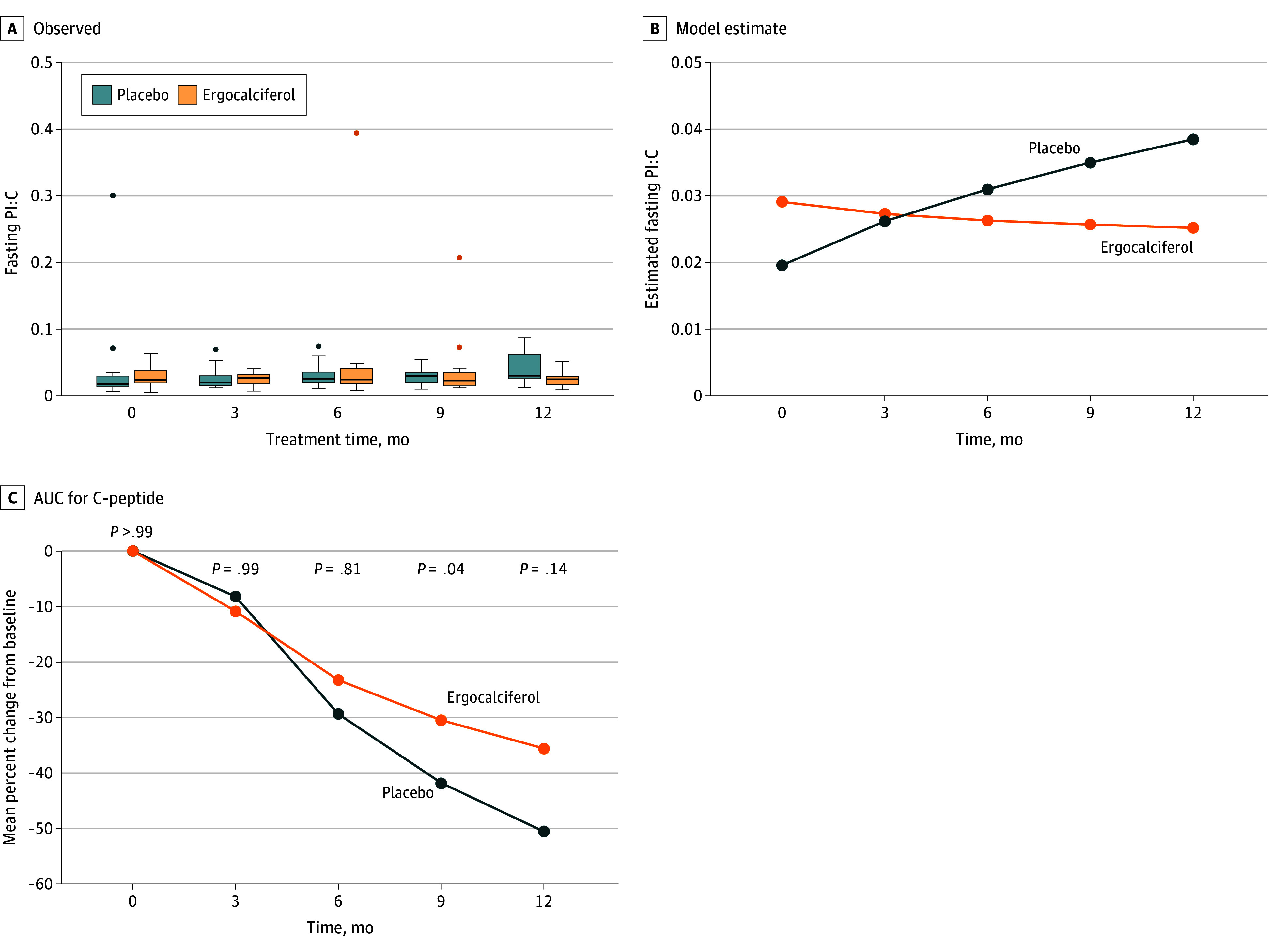
Fasting Proinsulin, Fasting C-Peptide, and Corresponding Proinsulin to C-Peptide Ratios (PI:Cs) A and B, Observed (A) and model-predicted (B) PI:Cs. Trends were generated from a repeated-measures generalized linear model of fasting PI:Cs. The total number of repeated-measures observations was 149 from 36 participants (18 per group). Three observed values were greater than 0.2; these were considered extreme outliers and were removed (in A). The remaining observations ranged from 0.005 to 0.087. The error distribution was normal, the link function was logarithmic, the repeated-measures correlation was unstructured, and the difference in trends between the 2 groups was significant (*P* = .01). C, Overall analysis of the trends showed that ergocalciferol significantly slowed the decline in percentage AUC C-peptide from baseline compared with placebo (*P* = .03).

## Discussion

In this study, ergocalciferol significantly decreased PI:C and slowed the decrease in %∆AUC C-peptide among youths with T1D. We previously showed that ergocalciferol significantly reduced temporal trends in HbA_1c_, IDAA_1c_, and TNF-α.^1^ Although this randomized clinical trial was limited by its single-center setting, the results suggest a protective action of ergocalciferol on β cells and possible mechanisms of action to prolong PR. Ergocalciferol’s ∆ effect size for β-cell protection (15%) is comparable to that of imatinib,^6^ verapamil,^4^ and other agents (15%-19.4%). Thus, vitamin D may be combined with other treatments (eg, teplizumab and baricitinib) to prolong PR.

## Notes

Supplement 1.
Trial Protocol

Supplement 2. **Trial Protocol.** Summary of IRB-Approved Amendments/Modifications to the Investigational Study Protocol With the Associated Dates

Supplement 3. **eFigure.** CONSORT Flow Diagram

Supplement 4.
Data Sharing Statement
