# Supplementary material for: Effect of Ergocalciferol on β-Cell Function in New-Onset Type 1 Diabetes: A Secondary Analysis of a Randomized Clinical Trial
Source: JAMA Netw Open. 2024 Mar 5;7(3):e241155. doi: 10.1001/jamanetworkopen.2024.1155 (PMC10915693; doi:10.1001/jamanetworkopen.2024.1155)
Supplement: Supplement 1. — Trial Protocol [file jamanetwopen-e241155-s001.pdf]

1) Title

Vitamin D Supplementation, Residual Beta-cell Function, and Partial Clinical Remission in Pediatric Type 1 diabetes: A 12-month Randomized, Double-Blind, Placebo-Controlled Trial

IRB Review History*

NA

2) Objectives*

1. To study how vitamin D affects the body during the honeymoon phase of type 1 diabetes using a randomized, double-blind, placebo-controlled trial design.

The study endpoints are:

(a) Change in the duration of the partial clinical remission (PCR) phase of T1D
(b) Change in the rate of decrease of residual beta cell function (RBCF) in T1D
(c) Change in the levels of glucagon-like peptide between the groups
(d) Change in the duration of PCR in subjects with high-risk T1D single nucleotide polymorphisms
(e) Change in glycemic control as measured by HbA1c.

Vitamin D is not being used for therapeutic intent for this trial, but for the evaluation of function of human system.

3) Background*

<u>Background:</u> The diagnosis of T1D is followed by a phase of PCR due to RBCF. During this early period in the natural history of T1D, endogenous islet function supports exogenous insulin therapy and enables the achievement of glycemic control with sub-physiologic insulin doses[1]. Recent studies indicate that approximately 50% of the β-cell mass may be viable at the time of diagnosis of T1D and that these β-cells persist for many months and even years following the diagnosis of T1D[2,3].

Preservation of RBCF is crucial for optimal diabetes care and outcome across the life history of T1D[4]. The landmark Diabetes Control and Complications Trial reported reduced incidence of long term diabetes-related end organ complication in patients with only modest levels of β-cell activity at entry into the trial[5]. Other studies show that endogenous insulin secretion in patients with T1D is associated with improved long-term glycemic control, reduced risk of severe hypoglycemia[3,6], reduced risk for the development of diabetic retinopathy[7] and a positive effect on statural growth in prepubertal children[8]. Regrettably, a recent study reported a low rate of PCR in children of <5years and adolescents of >12 years. This study also found that metabolic control was poorest in these young children, while the requirement for high doses of insulin to overcome insulin resistance decreased the duration of PCR in the adolescents[9]. Furthermore, a recent study reported that a variant of a principal T1D susceptibility gene, protein tyrosine phosphatase nonreceptor type 22 (PTPN22), the PTPN22 -1858T is associated with reduced residual β-cell function and poor metabolic control[10]. However, whether pharmacological interventions can modify the effect of these gene variants on PCR and RBCF is not well studied.

Similarly, the role of endogenous insulin secretagogues such as incretins on RBCF and PCR is not known in youth with T1D. Incretins are gastrointestinal hormones that stimulate insulin release after food ingestion. These are glucagon-like peptide 1 (GLP-1) and gastric inhibitory peptide (GIP). Though incretin-based

therapies are now being used to improve glycemic control and weight loss in patients with T2D[11], little is known about their effect on PCR, RBCF; and the factors that could prolong their effects. It is also unknown whether vitamin D treatment impacts GLP-1 secretion. These concepts may lead to effective and easily adoptable therapeutic modalities to prolong RBCF and the duration of PCR in youth with T1D.

Trials that investigated various modalities to prolong the duration of PCR led to differing conclusions[12-20]. Specifically, studies that examined the role of vitamin D on these parameters found that while the active form of vitamin D, calcitriol, demonstrated a protective effect on RBCF[20] in adult subjects with latent autoimmune diabetes of adults (LADA), two other studies in non-LADA patients with T1D, one of which enrolled children and adults, showed no effect of calcitriol on β-cell mass[15,16]. However, among the trials that investigated the effect of the precursor form of vitamin D, cholecalciferol, one trial in children of 6-12 years reported a non-statistically significant C-peptide decrease of 8% vs. 20% at 6 months in the vitamin D vs. placebo arm[14], while another trial in subjects of 7-30 years reported a statistically-significant C-peptide decrease of 14% vs. 46% after 18 months[12]. These slower rates of decrease in C-peptide values are comparable to the decreases in C-peptide achieved by immunotherapeutic intervention trials: 38% vs. 56%[17], 16% vs. 60%[18], 8% vs. 75%[19] at 18 months between the experimental and the placebo groups respectively. Taken together, these studies suggest that cholecalciferol supplementation may achieve a comparable protection of RBCF as immune-based therapies but without the toxic side effects[21,22].

However, **major limitations of these trials** include the use of suboptimal doses of vitamin D[12,14]; as well as the **lack of a standardized insulin treatment protocol** that addresses the confounding effect of concurrent insulin therapy on RBCF. This is important given that the cholecalciferol intervention trials reported no differences in HbA1c and insulin requirements[12,14] between the groups, while the immunotherapeutic trials reported improved HbA1c levels and decreased insulin requirements in their treatment groups[17,18]. To bridge this gap, and accurately determine how vitamin D affects the body during the honeymoon phase of T1D, we propose to standardize insulin therapy in both groups using an algorithm-based, self-directed, treat-to-target insulin regimen (TTIR) to address the confounding effects of differing insulin regimens on earlier trials.

Significance:

Type 1 diabetes (T1D) is a hyperglycemic syndrome caused by immune mediated destruction of β-cells of the pancreas that continues long after the diagnosis of the disease[12]. The high prevalence of poor glycemic control in children[23], and its attendant risk for lifetime debilitating chronic complications of diabetes have led to calls for innovative therapeutic interventions to prevent these chronic complications of diabetes mellitus. One of these maneuvers is to prolong the duration of partial clinical remission (PCR) by augmenting residual β-cell function (RBCF) in youth with newly diagnosed T1D. This is predicated on the report that approximately 50% of the β-cell mass may be viable at the time of diagnosis of T1D and that these β-cells persist for many months and even years following the diagnosis of T1D[2,3]. Studies that examined therapeutic agents for prolonging PCR have arrived at differing conclusions because of the severe side effect profile of the agents[21,22], insufficient dose of experimental agents[12,14], and more importantly, the non-standardization of insulin regimens[12,14,21,22] which confounded the effect of the experimental agents. The immunomodulatory approach is promising, but regimens tested to date lack sufficient benefit to justify the risk. Vitamin D supplementation, on the other hand, is safe and may slow T1D progression[12,14], but existing studies have caveats that prevent widespread implementation of this recommendation. For example, both trials used 2000 IU of vitamin D[12,14], and reported no changes in insulin requirements and HbA1c; while Gabbay et al[12] reported a significant difference in RBCF, Mishra et al[14] did not. Furthermore, the mechanisms of vitamin D effect on PCR and RBCF were not fully explored by both studies. This trial will address these gaps by employing an optimal dose of vitamin D in a randomized controlled trial with an in-built standardized insulin treatment regimen[24] to eliminate the confounding effect of non-standardized insulin treatment regimen on study outcomes. It will also examine mechanistic models of vitamin D action in prolonging PCR by examining

its effect on incretins, inflammatory markers, alterations in the levels of vitamin D binding protein (VDBP), and the alleviation of the deleterious effects of high-risk single nucleotide polymorphisms (SNPs).

The **rationale** for this study is to generate data to support or refute a recommendation for adjunctive vitamin D therapy to augment RBCF and prolong partial clinical remission (PCR) in patients with newly-diagnosed T1D.

This trial will add to existing knowledge by:
(a) determining the mechanism of vitamin D effect on PCR
(b) determining the role of vitamin D supplementation on the duration of PCR under standardized insulin treatment protocol
(c) determining the role of vitamin D supplementation in subjects with high-risk SNPs.

Ergocalciferol will be used for this trial. Ergocalciferol is vitamin D_2 whereas cholecalciferol is vitamin D_3. Both are analogs of the storage form of vitamin, 25-hydroxyvitamin D [25OHD], and are equipotent and undergo the same metabolic processes in human[25]. The only difference is that vitamin D_2 is the plant derived analog of 25OHD, while vitamin D_3 is the animal-derived analog of 25OHD. Whereas ergocalciferol is available in 50,000 IU formulation, cholecalciferol is not. This is why we are using ergocalciferol for this study.

The principal investigator has an extensive experience with ergocalciferol given his earlier open-label trial of 2000 IU of ergocalciferol in children of 10-18 years with psychiatric illness[26], and his current randomized controlled trial of ergocalciferol 50,000 IU per week in children, adolescents, and young adults with type 2 diabetes and nonalcoholic fatty liver disease [Docket #00002866]. In both the concluded trial and the ongoing trial, there were no reports of side effects such as hypercalcemia or hyperphosphatemia or their associated symptoms in these patients. Ergocalciferol is well tolerated in children, adolescents, and young adults.

4) Inclusion and Exclusion Criteria*

Inclusion criteria:

1. Age: 10-21 years.
2. Gender: Male and female
3. Tanner stage: II-V: to ensure the exclusion of prepubertal subjects.
4. T1D duration of <3 months (i.e., from first insulin injection) to ensure the inclusion of patients in PCR.
5. Presence of at least one diabetes-associated autoantibody.
6. Normal weight, overweight and obese subjects with T1D
7. Insulin therapy
8. Subjects on basal bolus regimen with an insulin-to-carbohydrate ratio
9. Fasting C-peptide level of >0.1 nmol/L (0.3 ng/mL)[19].

Exclusion criteria:

1. Subjects on weight altering medications, such as orlistat.
2. Subjects with eating disorder
3. Subjects on medications other than insulin that can affect blood glucose level.
4. Subjects with 25(OH)D levels of >70 ng/mL, as this may lead to vitamin D toxicity in the study subjects.
5. Subjects with major systemic diseases such as cardiac-, liver-, or kidney failure (requiring dialysis).
6. Subjects with recurrent diabetes ketoacidosis (>2 episodes since the diagnosis of T1D or in the preceding 3 months); or recurrent severe hypoglycemia (>2 episodes of hypoglycemia with altered level of consciousness, requiring a third party assistance in the preceding 3 mo).

7. Pregnant or breast-feeding female subjects. Even though vitamin D has no adverse effects on the health of a mother or developing fetus, we would want to limit this study to people who are not pregnant or breast-feeding.
8. The receipt of any investigational drug within 6 months prior to this trial.
9. Active malignant neoplasms.
10. Lack of internet access for uploading glucose data to MyCareTeam portal.

We will not enroll adults unable to consent, pregnant women, or prisoners.

5) Study-Wide Number of Subjects*

N/A.

6) Study-Wide Recruitment Methods*

N/A

7) Study Timelines*

Time	Event
April 2016	Obtain IRB Approval
June 2017	Start patient recruitment
June 2018	On-going data collection
June 2019	Study Completion, Data Analysis, Manuscript write up

8) Study Endpoints*

Primary endpoint:
1. Change in stimulated C-peptide concentration over time between the placebo and experimental groups
Secondary endpoints:
1. Change in HbA1c overtime during PCR between the placebo and experimental groups
2. Change in GLP-1, inflammatory markers, and vitamin D binding protein (VDBP) concentration over time between the placebo and experimental groups
3. Duration of PCR:
(a) Differences in the duration of PCR between the placebo and experimental groups
(b) Differences in the duration of PCR between the obese and normal-weight subjects
(c) Differences in the duration of PCR in subjects with high-risk SNPs who received vitamin D vs. placebo.

9) Procedures Involved*

Detailed procedures:

Subjects will be a part of the study for up to 13 months. Subject's endocrinologist will be notified of her participation in this study to ensure an open communication between the study staff and the endocrinologist.

METHODS AND ASSESSMENTS: VISIT PROCEDURES (See Table 1)

Protocol: This research study involves 8 visits (**Table 1)**: 2 screening visits, 1 visit during the run-in phase, and 5 study visits. In this 13 month study, 11 teaspoons of blood will be collected from each participant: one teaspoon at the second screening visit (V2), and 2 teaspoons at V4-V8. Subjects will undergo a one-month **run-in period** to screen for complications, to update patient education, and to assess subject's compliance with study protocol. Patients will continue on their pre-existing insulins. Subjects will not receive vitamin D or placebo during the run-in period. Subjects will be randomized to either arm of the study subjects will be randomized within 4 to 6 weeks of screening. Participants, who wish to do so, may continue to see their endocrinologists for their routine visits during their participation in the study.

VISIT 1: SCREENING VISIT

Consent: Consent and assent (for minors) will be obtained before being enrolled in the trial.
History: A detailed history of subject's medical condition will be taken to ensure eligibility for the study. This history will focus on patient's current medical diagnosis, past medical and birth history, social history. Family history of type 1 or type 2 diabetes, obesity, and related comorbidities will be documented. Subject's medications, supplements, diet and exercise plan will also be documented.
Physical Examination: All participants will undergo a detailed physical examination (PE) at Visits I (V1), then V4-V8. The aims of the PE are (1) to identify pathological features that could exclude patients from the study (2) to document subject's Tanner stage.
Urine Sample: A spot urine sample will be collected for pregnancy test in female subjects of child-bearing age who have achieved menarche. This will be repeated at V4-V8. Instructions for Mixed meal tolerance test (MMTT) will be provided at this visit.

Table 1. Summary of Study Protocol

Activities and Time points	V1	V2	V3	V4	V5	V6	V7	V8
	Screen 1	Screen 2	Run-In Start at time -4 weeks	Time 0 MONTH Randomization	M3	M6	M9	M12 EOS
Duration (hours)	1.5	2	2	2.5	2	2	2	2
Procedures								
Consent/Assent	X							
H&P	X			X	X	X	X	X
Vital signs	X			X	X	X	X	X
Anthropometric measures				X	X	X	X	X
MMTT instructions	X							
Screening test: C-peptide by MMTT		X						
Nutrition counseling			X			X		
Treat-to-target insulin regimen (TTIR)			X	X	X	X	X	
Blood glucose review and adjustments			X	X	X	X	X	
Research analytes: MMTT(c-peptide), C-reactive protein, 25OHD, VDBP, GLP-1, HbA1c, Ca, PO4, IL-1β, FGF21, TGF-β1,IL-4, IL-12, IL-2,TNF-α				X	X	X	X	X
Safety labs: Ca, PO4, 25OHD, urine Ca **These labs will also be collected at 4 and 8 weeks following randomization.**				X	X	X	X	X
Blood collection for SNPs				X				
Urine collection: VDBP, Urine Ca				X	X	X	X	X
Urine pregnancy test	X			X	X	X	X	
Run-In phase			X					
Adverse events		X	X	X	X	X	X	X
Dispense insulin and supplies			X	X		X	X	
Dispense ergocalciferol/placebo				X	X	X	X	
Collect ergocalciferol/placebo					X	X	X	X
Study diaries				X	X	X	X	
Follow up phone calls*								

* Subjects will be followed by weekly phone calls for 4 weeks until the time of randomization; and then once every month till the end of their participation in the study.
V=visit, M= month, EOS=end of study, MMTT= mixed meal tolerance test, TTIR= treat-to-target insulin regimen

VISIT 2: SCREENING VISIT FOR MIXED MEAL TOLERANCE TEST (MMTT)

Screening Laboratory Test: MMTT For Stimulated C-peptide Estimation. This test, aimed at stimulating endogenous C-peptide release, will be performed between 7 and 10AM following an overnight fast, with no injection of bolus insulin in the preceding 6 hours. Boost (formerly Sustacal, Mead Johnson, Evansville, IN, USA), at a dose of 6 mL/kg (maximum 360 mL), will be ingested in less than 10 minutes. Boost is a creamy, tasty, healthy drink that contains 26 vitamins and minerals, 3g fiber, and 10g of high-quality protein in each 8 fl oz bottle. Per the DCCT protocol, blood draws will be obtained for baseline glucose and C-peptide, and for 90-min post mixed-meal C-peptide and glucose estimation[27].

Even though this trial involves a standard fasting protocol which is defined as the lack of ingestion of calories for 8-12 hours, participants will not be at risk of hypoglycemia as they will not receive their short-acting insulin on the morning of the test until after the blood draws when they will then inject their short-acting insulin and eat a meal. They will also not be at risk for hyperglycemia as their bedtime long-acting insulin is titrated to maintain normal glucose levels when patient is not eating any calories.

VISIT 3: RUN-IN PHASE START AT -1 MONTH

*Run-In Phase: P*articipants will be started on treat-to-target insulin regimen (TTIR) on their existing insulins. All participants will be instructed on the modalities of TTIR and be given a **summary card** (Appendix 1) to familiarize themselves with the regimen. The PI and/or a certified diabetes educator (CDE) will review TTIR with the subjects at this visit. The basal-bolus regimen, on which the treat-to-target insulin regimen is based, is the standard treatment protocol for type 1 diabetes in children and adults, and is based on the results of the landmark Diabetes Control and Complications Trial which showed an improved glycemic control in subjects on basal bolus regimen compared to those on conventional therapy[5]. The only difference from routine diabetes management is that the patient and his or her parent/guardian will take an average of fasting blood glucose every third day and use that figure to titrate the dose of the long-acting insulin in patients on multiple daily injections, or the overnight basal rate for those who are using the insulin pump.

Glycemic control during the run-in-phase: A 1-month run-in period will precede the interventional phase of the study. All subjects will continue on their pre-existing insulins This will be followed by weekly phone calls by study staff and close monitoring of blood glucose levels while on their insulin regimen to ensure normoglycemia as shown in Tables 2 and 3 below. Parents will be advised to upload their child's glucose data in the MyCareTeam software portal weekly for study staff review and recommendation of insulin dose adjustment if necessary. In addition to the MyCareTeam upload, they can also fax a copy of their child's glucose log book to the study staff for review. This will enable the study team keep abreast of blood sugar trends in case of software system failures. All participants will return to the clinic at the end of one month (from the time of initiation of treat-to-target insulin regimen) for the randomization visit (Visit 4).
Subjects will be required to check their blood glucose at 3AM two times every week during this trial; and also to check their 3AM blood sugar for 2 nights in a row after any changes to their long-acting insulin.

Insulin Treatment Protocol:

Standardized Multiple Daily Insulin Injections Protocol: To ensure uniformity of insulin administration during the study, participants and their caregivers will use a self-directed titration algorithm of (-1)-0-(+1)[24] scale (Table 2) to adjust the dose of the subjects' long-acting insulin every 3rd day at bedtime to maintain fasting plasma glucose (FPG) in the normal range as shown in Table 3 below. Specifically, subjects will be advised to

increase their long-acting insulin (LAI) dose by 1 unit if the average of the 3 prior FPG recordings is >120 mg/dL; reduce their LAI dose by 1 unit if the average of 3 FPG readings is <90 mg/dL, and make no change to the LAI insulin dose if the average of 3 FPG values is between 90-120 mg/dL.

Standardized Protocol for Insulin Pump Therapy: Under parental supervision, patients on continuous subcutaneous insulin infusion (insulin pump) will similarly use a self-directed titration algorithm of (-10%)-0-(+10%) scale to adjust their overnight basal rate every 3rd day at bedtime to maintain fasting plasma glucose (FPG) in the normal range as shown in Table 2. Specifically, subjects will be advised to increase their overnight basal rate by 10% if the average of the 3 prior FPG recordings is >120 mg/dL; reduce their overnight basal rate by 10% if the average of 3 FPG readings is <90 mg/dL, and make no change to their overnight basal rate if the average of 3 FPG values is between 90-120 mg/dL.

Insulin to carbohydrate ratio: This will be adjusted as in routine clinical care as outlined in the Summary Card on Table 2: **Bolus insulin adjustment plan.**

Table 2: Titration Algorithm for Both Long-acting Insulin Analog and Overnight Basal Rate

Titration Algorithm for Both Long-acting Insulin (LAI) Analog or Overnight Basal Rate	
Average value of fasting plasma glucose for 3 consecutive days	Recommended long-acting insulin dose adjustments
<5.0 mmol/L (90 mg/dL)	• **Non-pump users**: subtract 1 units from the total dose of LAI<br>• **Pump users**: decrease overnight basal rate by 10%
5.0-6.7 mmol/L (90 – 120 mg/dL)	• no adjustments
>6.7 mmol/L (120 mg/dL)	• **Non-pump users**: add 1 units to the total dose of LAI<br>• **Pump users**: increase overnight basal rate by 10%

Table 3: Summary of Daily Plasma Glucose Goals

Time	Before breakfast	Before lunch or dinner	Before bedtime	2 hours after a meal	At 3AM
Glucose level (mg/dL)	90-120	80-130	> 100	< 220	> 100

Post-prandial glycemia will be maintained with adjusted doses of short-acting insulin for meal-time boluses using insulin to carbohydrate ratio and correction factor[28]. The concept of an algorithm-based, patient-directed standardization protocol for insulin delivery is innovative as no other trial to augment RBCF and prolong PCR in youth has employed this technique. The PI is well versed in the application of this protocol[24].

Follow Up Phone Calls: Subjects will be followed by weekly phone calls for 4 weeks until the time of randomization. These phone calls will focus on review of glucose data, insulin dose adjustments, and a review of the implementation of TTIR protocol. Participants needing further one-on-one instructions will be invited for a clinic visit before randomization.
These phone calls are safety checks and are required by the protocol, and will be documented by the study team on case report forms. The protocol specific window for these phone calls is 2 weeks from the prior phone call. This ensures that subjects, who were not reached in the first week, can still be called in the following week to ensure that the study team is compliant with the protocol specifications.

Nutrition Counseling: Participants will receive nutrition counseling from a registered dietician in the first 2 mo of the trial, and then at 6-8 mo. The counseling will focus on subject’s food choices and recommendations for a healthy diet based on MyPlate: each meal should consist of 50% vegetables and fruits, 25% carbohydrates, and 25% protein; with additional servings of water and dairy.

VISIT 4: BASELINE/RANDOMIZATION INTERVENTION STUDY VISIT AT 0 MO: Following the completion of the run-in phase, subjects will return for the randomization visit after an overnight fast for the initial intervention study blood draw.
Vital Signs: Blood pressure and pulse will be measured using an electronic sphygmomanometer.
Anthropometric Measurements: Weight, height and waist circumference will be measured in all participants at baseline, 3mo, 6 mo, 9 mo, and 12 months. Weight will be measured using an electronic scale with patient wearing under-garments and a hospital gown. Values will be expressed to the nearest 0.1 kg. Height will be measured using a wall-mounted stadiometer. Waist circumference will be measured to the nearest 0.1 cm at the superior border of the iliac crests using a standard measuring tape. All measurements will be done in triplicates and the average taken. Body mass index will be derived from the weight and height data.
Blood Samples: A venous catheter will be used to obtain blood sample for the analytes listed on **Table 1** under Research Analytes. Then, MMTT will be conducted as detailed on Visit 2. These analytes will be redrawn at 3, 6, 9, and 12 months. All samples will be spun upon collection using a centrifuge and the serum collected and stored at -70 degrees until assay. Whole blood sample will be obtained for HbA1c estimation using HPLC assay technique.
Urine Sample: A spot urine sample will be collected for baseline VDBP, which will be repeated at V5-V8.
SNP Genotyping and Research Assays: T1D risk-SNPs with odds ratio >1.5 (loci: DR3, DR4-DQ8, HLA-A24, HLA-B5701, HLA-DRB1-15, PTPN22, INS and IL2RA)[29] will be genotyped in triplicate using validated Taqman probes on a BioRad PCR machine. GLP-1 and inflammatory markers will be measured on baseline (inflammatory markers) or baseline and 90-minute (GLP1) DPP4-inhibitor-treated blood samples from MMTTs in duplicate using ELISA kits from Millipore. GLP-1 and inflammatory markers will be measured at V5-V8.

Randomization: Subjects will be randomized to either ergocalciferol or placebo. Ergocalciferol and placebo will be prepared as identical capsules by Boulevard Pharmaceutical Compounding Center[24]. Each pill will be of standard capsule size 00# which measures 20.1±0.35 mm in length and 7.66±0.02 mm in diameter.
Rationale for Treatment: For this trial, the study group will receive ergocalciferol 50,000 international units (IU) once weekly for 2 months, and then once every other week to keep serum 25(OH)D concentration between 20-100 ng/dL. This dose is below the standard tolerable upper intake level for vitamin D for people >9 yr [30] and thus unlikely to lead to vitamin D toxicity. This dosing regimen will also ensure an early rise in the serum 25(OH)D concentration.

Randomization will be conducted by the Investigational Drug Services (IDS), UMMS, using a randomization scheme generated by Dr. Barton. Randomization will be 1:1 (ergocalciferol: placebo) and will use a permuted block design with blocking for every 2 or 4 subjects (at random). IDS will maintain blinding information and PI will contact IDS for emergency unblinding. Twenty subjects will be randomized to each arm of the trial as follows: 40 subjects will be stratified by BMI criterion into 20 normal-weight (BMI<85th percentile), and 20 overweight/obese subjects (BMI >85th percentile). Ten subjects from each BMI group will receive ergocalciferol while the other 10 will receive placebo thus allowing for stratification by both BMI and treatment modality. Subjects will receive a pill-counting dossette for monitoring of compliance during follow up visits. Group allocation will remain hidden to all study staff and patients until all data have been collected and analyzed.

Following randomization, participants will continue on TTIR, with meal-time insulin to carbohydrate ratio and correction factor to ensure euglycemia between meals. The adequacy of each aspect of therapy will be confirmed by an ongoing monitoring system as described by Bode et al[28], and summarized in Tables 5 and 6.

Follow-Up Phone Calls: Following randomization, subjects will be contacted once every month until the end of participation in the trial. The subject of the calls will be to identify adverse effects, assess compliance and accuracy of insulin titration, and make recommendations.
These phone calls are safety checks and are required by the protocol, and will be tracked by the study team. The protocol specific window for these phone calls is 2 months from the prior phone call. This ensures that subjects, who were not reached in the prior month, can still be called in the subsequent month, to ensure that the study team is compliant with the protocol specifications.

Glucose Data Collection: This trial will use the MyCareTeam software for comprehensive glucose data capture. MyCareTeam[31] is a secure software portal/system in widespread clinical use in the UMass adult and pediatric diabetes clinics that enables patients to directly upload their glucose data to the hospital's electronic medical record system for immediate review by study staff. Fasting and non-fasting plasma glucose data will be obtained by the patient and/or caregiver by self-monitoring of patient's capillary blood glucose (SMBG) using the Precision Xtra Blood Glucose Monitoring System (Abbott Diabetes Care, Alameda, CA, USA) or via the subjects insulin pump. All participants will receive a Study Summary Card containing a synopsis of the daily study guidelines. Parents and study subjects will routinely upload subjects' glucose data to the UMass MyCareTeam [31] website every 4 weeks for review by the study staff. Along with data recorded by families in the titration algorithm sheets, the MyCareTeam software will provide comprehensive data on hypoglycemic events, fasting and non-fasting plasma glucose levels, and compliance rate with capillary blood glucose monitoring which will ensure a comprehensive glucose data analysis. Following randomization, participants will return to the clinic at 3, 6, 9 months for interim visits, and at 12 months for study conclusion.

Study Diary: All study subjects will be given a study diary for the purposes of documenting ergocalciferol/placebo administration and monitoring of compliance. Subjects should put a check mark on the correct date in the diary right after taking their ergocalciferol or placebo. The patients should bring in the diaries for a review by the study staff at every visit following randomization.

VISIT 5: INTERVENTION STUDY INTERIM VISIT AT +3 MO: Subjects will return to the clinic three months after randomization to either ergocalciferol or placebo, while continuing on TTIR. This visit will involve a repeat assessment of vital signs, anthropometric measurements, physical examination, review of dossettes, MMTT, and fasting laboratory investigations as detailed for Visit 4.

VISIT 6: INTERVENTION STUDY VISIT AT +6 MO
Subjects will undergo evaluations as detailed in the section on Visit 4, in addition to a nutrition evaluation.

VISIT 7: INTERVENTION STUDY VISIT AT +9 MO
This visit will involve assessment of vital signs, anthropometric measurements, physical examination, review of dossettes, MMTT and fasting blood and urine collection as detailed in the section on Visit 4.

VISIT 8: STUDY CONCLUSION AT +12 MO
This visit will involve a final assessment of vital signs, anthropometric measurements, physical examination, review of dossettes, MMTT and fasting blood and urine collection as detailed in the section on Visit 4. The discharge history and physical examination will focus on identifying any adverse effects from the study, and to establish endpoint laboratory and anthropometric data. Ergocalciferol/placebo will be discontinued at this visit. The TTIR will end at the time of study completion, but participants will be encouraged to discuss continuing TTIR with their endocrinologists.

10) Data and Specimen Banking*

N/A – No banking for future research.

11) Data Management*

Anthropometric data, urine, and blood samples will be collected from subjects for research purposes only.

Only research team members will have access to subject identifying information. Hard copy data will be transferred (hand-carried by a research team member) from the CRC to the Department of Pediatrics for data entry and analysis. Each patient will be identified by a study ID number. A list of subject identifying information linked to the study ID numbers will be kept in a computer file which is password protected and a hard copy kept in a locked file in the trial coordinator's locked office in the Clinical Research Center, AC1-044, University of Massachusetts Medical School.

STATISTICAL CONSIDERATIONS:

All analyses will be overseen by Dr. Barton, Professor of Biostatistics at UMMS, and performed by analysts at the Quantitative Methods Core.

Sample size and power calculation: This trial's sample size was based on establishing a stable estimate (with a 95% confidence interval) for the difference in C-peptide between the two treatment groups. Based on published data[16], group sample sizes of 13 in each arm, will produce a two-sided 95% confidence interval with a distance from the difference in means to the limits of 0.081 nmol/l when the estimated standard deviation is 0.10 in each group; i.e., if the estimated difference in C-peptide between the two treatment groups is 0.12 nmol/L, the 95% confidence interval will be ± 0.081. Calculated sample size will be increased to 40 to enable further analysis by BMI strata and to compensate for attrition.

Data Management

Data will be entered using the REDCap system (Vanderbilt University) as implemented at the University of Massachusetts Medical School, a member of the REDCap Software Consortium, using SQL Server as the underlying database structure. We will program the system for a single entry with validation rules at the time of entry and comprehensive edits conducted after the data have been submitted to the main data base. These edits will check for validity, consistency, and normal range values. Edit queries will be generated and resolved by clinic staff with corrections posted to the database through the REDCap system, which enforces an audit trail for all changes. The main study database will be stored on a secure server in the University of Massachusetts HIPAA-compliant data center with daily back-up. For analysis, data will be exported from the REDCap system as SAS data sets and merged within SAS to create the official analysis files for the study. All reports and analyses will be generated from these files using the latest version of SAS (currently SAS 9.2). Data files (and accompanying SAS programs) that are used for reports, presentations, or publication will be archived as required past the end of the study.

The REDCap system will be programmed and maintained by staff at the Quantitative Methods Core at the U Mass Medical School while data entry will be performed by staff in the Department of Pediatrics at the UMass Medical School.
Data entered into REDCap is password protected and access is limited to the research staff.

Storage:
Specimens will be stored at -80 C in the Clinical Research Center, Room AC1-044, in the ACC Building, UMMS, until study completion. All stored samples will be maintained solely for use in this research project.
Only the PI and members of his research team will have access to stored specimens (blood and urine samples).

Data collected as part of this study will be stored for 3 years following the completion of statistical analysis.

12) Provisions to Monitor the Data to Ensure the Safety of Subjects*

To monitor for safety, calcium, phosphorus, and 25(OH)D will be drawn at 4 and 8 weeks after randomization, and visits 4-8. Urine calcium will be obtained with other safety labs at visits 4-8. The normal levels of these analytes are: calcium 8.7 -10.7 mg/dL, Phosphorus 3.5-5.9 mg/dL, and 25(OH)D 30-100 ng/mL. Subjects with elevated calcium, phosphorus, or 25(OH)D will be withdrawn from the study and not restarted on study pills.

Safety above all else is our concern. We will ensure that all study patients are comfortable during each assessment. In addition, we will be extremely attentive to any potential emotional or behavioral challenge that might limit a subject's participation in the study.

Dr. Amanda Angelescu, a pediatric endocrinologist who is not connected with this study will serve as the Data Monitoring Officer. She will oversee the overall safety of subjects during the duration of this study. Dr. Angelescu is an experienced endocrinologist and diabetologist with extensive clinical and research experience in the management of children and adolescents with T1D.

Reports on adverse effects will be collected at study visits, and also by telephone in between visits. Research staff will contact subjects twice in the first month, and then once every 2 months throughout the study. Each subject will be contacted 3 additional times if they are not able to be reached at the first call.

During the phone calls the subject or his/her parent(s) will be asked about his or her compliance with the study; any side effects that could be related to elevated calcium levels such as weakness, constipation, excessive urination.

Data on adverse effects will be entered in a database and sorted by treatment group. The investigators will review these data once every month and present this report to the Data Monitoring Officer, who will forward such reports to the UMass IRB. Any events which meet the UMMS prompt reporting requirements will be reported to the UMMS IRB within 5 business days of the PI becoming aware of the information.

13) Withdrawal of Subjects*

A subject must be withdrawn from the study if any of the following applies:

1. Pregnancy or intention of becoming pregnant
2. Allergic reaction to vitamin D or placebo
3. Subject decides to withdraw from the study
4. Subject may also be withdrawn from the trial at the discretion of the Investigator if judged non-compliant with trial procedures or due to a safety concern
5. Subjects with elevated calcium, phosphorus, or 25(OH)D will be withdrawn from the study and not restarted on study pills.

In addition, any enrolled subject who is found to be ineligible based on the inclusion and exclusion criteria outlined during the screening visit or any visit thereafter will be withdrawn from the study.

14) Risks to Subjects*

Some children, adolescents, and young adults may become embarrassed when given a physical examination or asked detailed questions about their type 1 diabetes.

Risk of venipuncture:
The risks of having blood drawn include slight pain when the needle is inserted. Subjects may develop a harmless black and blue mark, and they arm may be sore. Occasionally, some people feel dizzy or lightheaded when blood is drawn. They may become sweaty, feel cold or tingly, and may faint or throw up. Risks that are possible but unlikely include infection, nerve damage, and puncturing an artery instead of a vein.

As a result of participating in this study, subjects randomized to the intervention group will receive ergocalciferol 50,000 IU every week for 2 months, and then once every other week for 10 months. The standard management of vitamin D deficiency in the study age group is 50,000 IU of ergocalciferol once weekly for 6 weeks, followed by a recheck of 25(OH)D level and maintenance ergocalciferol 50,000 IU once every two weeks. This is a very high dose of vitamin D.

There is a small risk of a Vitamin D overdose in this trial. This may manifest as hypercalcemia or hyperphosphatemia. The associated symptoms include stomach upset (example: nausea, vomiting, change in bowel movements), weakness, weight loss, confusion and heart palpitations. Calcium and phosphorus levels will be checked at each visit following randomization to ensure normal serum calcium and phosphorus levels. Subjects with elevated calcium, phosphorus, or 25(OH)D will be withdrawn from the study and not restarted on study pills.

Risk of hypercalciuria (elevated calcium in the urine):
There is a minimal risk of hypercalciuria in this study. Urine calcium and creatinine will be monitored throughout the study to ensure normocalciuria.A normal reference interval for random urine calcium (mg/dL):urine creatinine (mg/dL) ratio is <0.14. Values exceeding 0.20 are found in patients with hypercalciuria[32].

Allergic reactions to ergocalciferol are extremely rare, but possible manifestations of an allergic reaction will include signs and symptoms such as rash, itching/swelling, severe dizziness, and trouble breathing.

Fasting

Even though high blood sugar levels and/or low blood sugar levels are not anticipated, they are still potential risks of the study. Hyperglycemia will be unlikely to occur as the patient will have his 24-hour long-acting insulin on board. Hypoglycemia is also unlikely to occur as the subject will not receive any insulin until the end of testing when he/she will be free to eat.

However, if the subject experiences hypoglycemia, he/she may feel hungry, shaky, weak and dizzy. The patient's parent or study staff will check the patient's blood sugar, and if low (<70 mg/dL), the patient will be managed based on the American Diabetes Association guidelines for managing hypoglycemia which is to administer 15g of carbohydrate, wait for 15 minutes, and recheck blood sugar; and retreat with 15g if it is blood glucose is still low.

If patient experiences hyperglycemia, he/she may feel no symptoms, or he/she could feel slightly weak. The subject's parent or study staff will his/her blood glucose and if >250 mg/dL, will administer a correction insulin dose based on the subject's insulin regimen.

As with all studies, there is a chance that data could be linked back to a subject's true identity, and so there is a risk of psychological and social stigma that could adversely impact the subject.

15) Potential Benefits to Subjects*

Subjects may benefit directly as follows:

Vitamin D Repletion: All participants in the experimental arm of the study will receive vitamin D supplementation during the study. This vitamin D repletion will confer all the benefits of vitamin D sufficiency on the subjects.

Monitoring and more frequent follow up visits: This will ensure the safety of the participants and may improve their adherence to their insulin regimen.

Medical Nutrition Therapy: Registered pediatric nutritionists will instruct the subjects on the state-of-the-art modalities of medical nutrition therapy. This will ensure a healthy eating habit and the maintenance of healthy weight and energy.

16) Vulnerable Populations*

Provisions are made for soliciting permission of the parents/ legal guardians of those persons who have not yet attained the legal age for consent.

We will not recruit from any other vulnerable populations.

The level of risk associated with this study is minimized as these children will continue on their standard insulin regimen and will only need to titrate their long-acting insulin doses, or overnight basal rates once every 3 days, to ensure normal fasting glucose levels; while also adjusting their short-acting insulin as in routine care to keep their blood glucose in the normal range.

Vitamin D is a supplement with proven safety profile which is well-tolerated by children.

The research team members have extensive experience in the use of vitamin D and TTIR in research studies in children.

17) Multi-Site Research*

NA

18) Community-Based Participatory Research*

NA

19) Sharing of Results with Subjects*

The following results will be shared with subjects and their families: end of study vitamin D status.

20) Setting

All subjects will be recruited from the Diabetes Center of Excellence of the UMass Memorial Medical Center.

Consenting and all study related procedures will be done in private exam rooms in the Clinical Research Center, located in the Ambulatory Care Center. No research related activities will be conducted outside of the Ambulatory Care Center.

21) Resources Available

Facilities:

The Ambulatory Care Center (ACC) Building is a new healthcare facility that serves several outpatient subspecialty clinics including the Pediatric Endocrinology and Diabetes Clinics.

On the first floor of the ACC Building is located the UMMS Clinical Research Center (CRC), which is a 2,600-square-foot ambulatory research site that provides a comfortable and accessible area for study participants. The CRC includes a waiting area; conference room for study initiation, monitoring visits, and educational meetings; four (4) staff offices; three workstations for visiting staff; four (4) exam rooms, and an infusion room with two recliners. It is staffed with an experienced, certified clinical research nurse manager, research nurse coordinators, regulatory specialist and a receptionist / research assistant. It provides a high quality research

environment for the coordination and management of clinical studies. A small laboratory equipped with centrifuges, freezer, and hematocrit machine is available to investigators and their staff, as well as an area for packaging and shipping clinical trial samples to meet current transport regulations. The CRC also has a secure area for short-term storage of study files and case report forms. The Clinical Research Center is under the direction of the Office of Clinical Research in the Office of Research, UMass Medical School.

Personnel:

The Diabetes Center of Excellence (DCOE) is staffed by experienced pediatric and adult endocrinologists and certified diabetes educators. The study staff will work with members of the DCOE team to ensure that the enrollment goal of 40 subjects is reached.

All of the pediatric endocrinologists and Certified Nurse Educators have multiple years of experience working with children and adolescents with T1D. Equally, the pediatric nutritionists have multiple years of experience working with children and adolescents.

Principal Investigator will be responsible for study design and statistical analysis. The PI is a board-certified pediatric endocrinology with extensive experience in research studies in children involving vitamin D and treat-to-target insulin regimen.

Principal Investigator and Co-investigators will be responsible for recruitment, consenting, obtaining medical and family history, conducting physical exams, as well as SAE/AE monitoring/ reporting.
The co-investigators include a board-certified internist with extensive experience in single-nucleotide polymorphism genotyping in diabetes mellitus, and processing of inflammatory markers; and a board-certified biostatician with extensive experience in the design and analysis of research studies in children. A sub-investigator will be a terminal degree holder whose role will be limited to identifying eligible subjects for this study.

Registered pediatric nutritionists will be responsible for instructing the subjects on state-of-the-art modalities of medical nutrition therapy.
<u>The counseling sessions will occur</u> in the first 2 mo of the trial, and then at 6-8 mo. The counseling sessions will focus on subject’s food choices and recommendations for a healthy diet based on MyPlate: each meal should consist of 50% vegetables and fruits, 25% carbohydrates, and 25% protein; with additional servings of water and dairy.

Nurse coordinators will be responsible for recruitment, consenting, obtaining medical and family history, phlebotomy, monitoring vital signs/ nursing assessments, maintain regulatory documents, CRF completion and query resolution, and IRB communications.

A Research Nurse Coordinator in the Clinical Research Center now serves as the primary study coordinator. The other CRC nurses will act as back up and will be trained to carry out study visits in the absence of the primary coordinator. Each Nurse Coordinator in the Clinical Research Center has greater than 10 years’ experience in clinical research. Weekly team meetings are held to review studies on an ongoing basis.

All study staff will have completed the required CITI training.

22) Prior Approvals

This study is entirely covered under the blanket of the CRC's IBC prior approval.

23) Recruitment Methods

Participants will be recruited from the outpatient clinic of the UMass Memorial Health Center (UMMHC).

Potentially eligible children and adolescents will be identified for the study by pediatric endocrinologists. The endocrinologist will then review the subject's medical records to ensure that the patient is eligible for the study. Once identified, the subject's pediatric endocrinologist will discuss this study with the potential subject and his/her parent(s) or guardian(s). If they are interested in learning more, they will be contacted by the s study staff, in this case an endocrinologist or a research nurse coordinator, to go over the details of the study to determine whether they are interested in participating in the study.

Prospective study patients who are under the care of the study investigators will be recruited directly to the study. Prospective study patients who are under the care of endocrinologists who are not part of the study team will be contacted by their treating endocrinologist and asked if it is okay to pass on their contact information to the study team for recruitment purposes.

The study staff will contact the prospective subject and/or his or her parents using a phone screening script that will be submitted to the IRB for review and approval prior to use after the study staff is given the okay by the treating endocrinologist to contact the subject.

No recruitment letters are needed for this study.

Patients who meet the above-outlined inclusion/exclusion criteria will be approached by a study team member during their regular clinic appointments. If they are interested in participating, a screening appointment will be arranged.

Subjects will be recruited from the outpatient pediatric clinic at the UMass Memorial Health Center (UMMHC). The UMMHC has a broad outpatient base and a thriving Diabetes Center for Excellence.

Recruitment:
Feasibility: Sixty to eighty patients of age <21yr are diagnosed with new-onset T1D at the Children Medical Center every year, and 30-35% of them are overweight or obese. Thus we have the patient population to carry out this study. The recruitment timeframe for this study is <12 months as we will be able to 'back recruit' eligible patients going through the honeymoon phase, and prospectively recruit newly-diagnosed patients during the trial.

Study Compensation:
This research study involves 8 visits (**Table 1**): 2 screening visits, 1 visit during the run-in phase, and 5 study visits.
Participants will receive $50 at the end of each visit. This payment will be made out in the form of a gift card to a bookshop. Participants will also be provided with a parking voucher at the end of each visit.

24) Local Number of Subjects

We plan to screen 120 subjects for this study. Of these, we expect to enroll 20 study patients, and 20 control subjects for the study.

25) Confidentiality

Only the research study team including the statistician and data manager will have access to the study information and data collected. The following procedures will be taken for protecting and limiting access to all the participating subjects' personal information and ensuring subject anonymity/confidentiality.

1. We will assign an ID number to each subject and associated data.
2. No personal identifying information will be contained on the data collection forms.
3. The list that links identifiers with the study ID numbers will be kept in a password-protected computer file and in hard copy in a locked file by the study coordinator in the Clinical Research Center, AC1-044. Reporting information will be done in aggregate form or with qualitative illustration.
4. The University of Massachusetts firewall protects online data and is secured with daily data back-up.

Only research team members will have access to subject identifying information. Hard copy data will be transferred (hand-carried by a research team member) from the CRC to the Department of Pediatrics for data entry and analysis. Each patient will be identified by a study ID number. A list of subject identifying information linked to the study ID numbers will be kept in a computer file which is password protected and a hard copy kept in a locked file in the trial coordinator's locked office in the Clinical Research Center, AC1-044, University of Massachusetts Medical School.

26) Provisions to Protect the Privacy Interests of Subjects (HIPAA)

All subjects will be asked to sign a HIPAA consent form at the time of signing of the IRB consent or assent to allow us access to pertinent clinical information for the study.

27) Compensation for Research-Related Injury

If a subject is injured while on study, he/she is advised to seek treatment and contact the study doctor as soon as possible. The University of Massachusetts Medical School does not provide funds for the treatment of research-related injury.

If a subject is injured as a result of his/her participation in this study, treatment will be provided. However, the subject or his/her insurance carrier will be expected to pay the costs of the treatment. No additional financial compensation for injury or lost wages will be made available. As stated in the consent form, the subject does not give up any of his/her legal rights by signing this form.

28) Economic Burden to Subjects

There will be no additional cost to the subjects. The study will cover the cost of vitamin D or placebo used in the trial.

29) Consent Process

Both parents must give their permission for children unless one parent is deceased, unknown, incompetent, or not reasonably available, or only one parent has legal responsibility for the care and custody of the child as required by the federal regulations at 45 CFR 46.408(b).
All children ages 10 to 15 will sign an approved assent form detailing the study procedures to document their assent. Children 16 and 17 years old will sign the consent form to document their assent. Participants 16 years and older will be given a consent form. The consent and assent forms will be read to the subjects and their parents/guardians. They will then be given the time they need to review the form and ask questions about the study. When the subject and his/her family are completely satisfied about their participation in the study, they will be invited to sign the forms in the presence of a study investigator or research nurse and a witness.

Only individuals who are fluent in English will be enrolled. However, there may unexpected situations where a non-English speaking subject will be enrolled in this study. In such a case, we will only enroll the subject if there is an IRB approved short form available in the subject's language. The PI will follow the UMMS Investigator Guidance for Informed Consent (HRP-802) and UMMS Investigator Guidance for Documentation of Informed Consent (HRP-803). These stipulate that the consenting process will require the services of an interpreter who is fluent in English and the subject's language, an independent witness who is fluent in English and the subject's language (the interpreter and witness may be the same individual), and a member of the study staff. When the subject and his/her family are completely satisfied about their participation in the study, they will be invited to sign the forms in the presence of a study investigator or research nurse. We will not enroll a child if there is any sign of unwillingness.

Please see #21 for a description of where the consent process will take place.

30) Process to Document Consent in Writing

We will document the consent in writing, in the form of a signed Informed Consent Form. We will be following the UMMS Investigator Guidance for Documentation of Informed Consent (HRP-803). This means that for non-English speaking subjects, the subject, the witness, and person obtaining consent will each sign the summary (i.e., the long consent form) and the short form.

31) Drugs or Devices

No drug is being tested in this study.

Storage and Administration: Both the vitamin D capsules and placebo (containing microcrystalline cellulose) will be compounded by Boulevard Pharmaceutical Compounding Center, Worcester, Massachusetts, and supplied to the Investigational Drug Services (IDS), University of Massachusetts for storage and dispensing. Only the IDS staffers have the authority to dispense ergocalciferol or placebo as part of the randomization and ongoing dispensing of study drugs. The PI, Dr. Nwosu, has an ongoing relationship with both the Boulevard Pharmaceutical Compounding Center and IDS as part of his ongoing randomized controlled trial.

References:

1. Retnakaran R (2015) Novel Strategies for Inducing Glycemic Remission during the Honeymoon Phase of Type 2 Diabetes. Can J Diabetes.

2. Oram RA, Jones AG, Besser RE, Knight BA, Shields BM, et al. (2014) The majority of patients with long-duration type 1 diabetes are insulin microsecretors and have functioning beta cells. Diabetologia 57: 187-191.

3. Sorensen JS, Johannesen J, Pociot F, Kristensen K, Thomsen J, et al. (2013) Residual beta-Cell function 3-6 years after onset of type 1 diabetes reduces risk of severe hypoglycemia in children and adolescents. Diabetes Care 36: 3454-3459.

4. Max Andersen ML, Hougaard P, Porksen S, Nielsen LB, Fredheim S, et al. (2014) Partial remission definition: validation based on the insulin dose-adjusted HbA1c (IDAA1C) in 129 Danish children with new-onset type 1 diabetes. Pediatr Diabetes 15: 469-476.

5. Steffes MW, Sibley S, Jackson M, Thomas W (2003) Beta-cell function and the development of diabetes-related complications in the diabetes control and complications trial. Diabetes Care 26: 832-836.

6. Sherry NA, Tsai EB, Herold KC (2005) Natural history of beta-cell function in type 1 diabetes. Diabetes 54 Suppl 2: S32-39.

7. Nakanishi K, Watanabe C (2008) Rate of beta-cell destruction in type 1 diabetes influences the development of diabetic retinopathy: protective effect of residual beta-cell function for more than 10 years. J Clin Endocrinol Metab 93: 4759-4766.

8. Bizzarri C, Benevento D, Patera IP, Bongiovanni M, Boiani A, et al. (2013) Residual beta-cell mass influences growth of prepubertal children with type 1 diabetes. Horm Res Paediatr 80: 287-292.

9. Bowden SA, Duck MM, Hoffman RP (2008) Young children (<5 yr) and adolescents (>12 yr) with type 1 diabetes mellitus have low rate of partial remission: diabetic ketoacidosis is an important risk factor. Pediatr Diabetes 9: 197-201.

10. Petrone A, Spoletini M, Zampetti S, Capizzi M, Zavarella S, et al. (2008) The PTPN22 1858T gene variant in type 1 diabetes is associated with reduced residual beta-cell function and worse metabolic control. Diabetes Care 31: 1214-1218.

11. Hur KY, Lee MS (2015) New mechanisms of metformin action: Focusing on mitochondria and the gut. J Diabetes Investig 6: 600-609.

12. Gabbay MA, Sato MN, Finazzo C, Duarte AJ, Dib SA (2012) Effect of cholecalciferol as adjunctive therapy with insulin on protective immunologic profile and decline of residual beta-cell function in new-onset type 1 diabetes mellitus. Arch Pediatr Adolesc Med 166: 601-607.

13. Ataie-Jafari A, Loke SC, Rahmat AB, Larijani B, Abbasi F, et al. (2013) A randomized placebo-controlled trial of alphacalcidol on the preservation of beta cell function in children with recent onset type 1 diabetes. Clin Nutr 32: 911-917.

14. Mishra A, Dayal D, Sachdeva N, Attri SV (2015) Effect of 6-months' vitamin D supplementation on residual beta cell function in children with type 1 diabetes: a case control interventional study. J Pediatr Endocrinol Metab.

15. Walter M, Kaupper T, Adler K, Foersch J, Bonifacio E, et al. (2010) No effect of the 1alpha,25-dihydroxyvitamin D3 on beta-cell residual function and insulin requirement in adults with new-onset type 1 diabetes. Diabetes Care 33: 1443-1448.

16. Bizzarri C, Pitocco D, Napoli N, Di Stasio E, Maggi D, et al. (2010) No protective effect of calcitriol on beta-cell function in recent-onset type 1 diabetes: the IMDIAB XIII trial. Diabetes Care 33: 1962-1963.

17. Pescovitz MD, Greenbaum CJ, Krause-Steinrauf H, Becker DJ, Gitelman SE, et al. (2009) Rituximab, B-lymphocyte depletion, and preservation of beta-cell function. N Engl J Med 361: 2143-2152.

18. Herold KC, Gitelman S, Greenbaum C, Puck J, Hagopian W, et al. (2009) Treatment of patients with new onset Type 1 diabetes with a single course of anti-CD3 mAb Teplizumab preserves insulin production for up to 5 years. Clin Immunol 132: 166-173.

19. Ludvigsson J, Faresjo M, Hjorth M, Axelsson S, Cheramy M, et al. (2008) GAD treatment and insulin secretion in recent-onset type 1 diabetes. N Engl J Med 359: 1909-1920.

20. Li X, Liao L, Yan X, Huang G, Lin J, et al. (2009) Protective effects of 1-alpha-hydroxyvitamin D3 on residual beta-cell function in patients with adult-onset latent autoimmune diabetes (LADA). Diabetes Metab Res Rev 25: 411-416.

21. Chakhtoura M, Azar ST (2013) The role of vitamin d deficiency in the incidence, progression, and complications of type 1 diabetes mellitus. Int J Endocrinol 2013: 148673.

22. van Belle TL, Coppieters KT, von Herrath MG (2011) Type 1 diabetes: etiology, immunology, and therapeutic strategies. Physiol Rev 91: 79-118.

23. Willi SM, Miller KM, DiMeglio LA, Klingensmith GJ, Simmons JH, et al. (2015) Racial-ethnic disparities in management and outcomes among children with type 1 diabetes. Pediatrics 135: 424-434.

24. Nwosu BU, Maranda L, Cullen K, Greenman L, Fleshman J, et al. (2015) A Randomized, Double-Blind, Placebo-Controlled Trial of Adjunctive Metformin Therapy in Overweight/Obese Youth with Type 1 Diabetes. PLoS One 10: e0137525.

25. Biancuzzo RM, Clarke N, Reitz RE, Travison TG, Holick MF (2013) Serum concentrations of 1,25-dihydroxyvitamin d2 and 1,25-dihydroxyvitamin d3 in response to vitamin d2 and vitamin d3 supplementation. J Clin Endocrinol Metab 98: 973-979.

26. Nwosu BU MB, Maranda L, Ciccarelli C, Reynolds D, Curtis L, King J, Frazier JA, Lee MM. (2011) A potential role for adjunctive vitamin D therapy in the management of weight gain and metabolic side effects of second-generation antipsychotics. Journal of Pediatric Endocrinology and Metabolism (in press).

27. (1998) Effect of intensive therapy on residual beta-cell function in patients with type 1 diabetes in the diabetes control and complications trial. A randomized, controlled trial. The Diabetes Control and Complications Trial Research Group. Ann Intern Med 128: 517-523.

28. Bode BW, Davidson, P.C., editor (1995) The Insulin Pump Therapy Book. Los Angeles: Minimed Technologies.

29. Oram RA, Patel K, Hill A, Shields B, McDonald TJ, et al. (2016) A Type 1 Diabetes Genetic Risk Score Can Aid Discrimination Between Type 1 and Type 2 Diabetes in Young Adults. Diabetes Care 39: 337-344.

30. Medicine Io (2010) Dietary Reference Intakes for Calcium and Vitamin D; Ross AC, editor. Washington, DC: National Academy Press.

31. Smith KE, Levine BA, Clement SC, Hu MJ, Alaoui A, et al. (2004) Impact of MyCareTeam for poorly controlled diabetes mellitus. Diabetes Technol Ther 6: 828-835.

32. Foley K (2010) Urine Calcium: Laboratory Measurement and Clinical Utility. Lab Med 41: 683-686.
