# Supplementary material for: Effect of Ergocalciferol on β-Cell Function in New-Onset Type 1 Diabetes: A Secondary Analysis of a Randomized Clinical Trial
Source: JAMA Netw Open. 2024 Mar 5;7(3):e241155. doi: 10.1001/jamanetworkopen.2024.1155 (PMC10915693; doi:10.1001/jamanetworkopen.2024.1155)
Supplement: Supplement 2. — Trial Protocol. Summary of IRB-Approved Amendments/Modifications to the Investigational Study Protocol With the Associated Dates [file jamanetwopen-e241155-s002.pdf]

Summary of IRB-approved Amendments/Modifications to the Investigational Study Protocol with the Associated Dates **15SEPT2021**

Study title: Vitamin D Supplementation, Residual Beta-cell Function, and Partial Clinical Remission in Pediatric Type 1 diabetes: A 12-month Randomized, Double-Blind, Placebo-Controlled Trial

This study was originally approved by the University of Massachusetts Institutional Review Board (IRB) on 5/27/2016.

All amendments reviewed and approved by the University of Massachusetts IRB are listed below:

On 3/8/2017, study protocol was updated with Section 2.0 Funding Sources: the study team uploaded a copy of the grant, and updated study documents to allow the NIH and FDA to provide oversight for the trial.

Submission of study revision 1 (9/25/17 & 12/04/2017): minor changes to study personnel, study personnel responsibilities and clarification of a study flyer to be used for recruitment purposes.

The following changes were made to the study Investigational Study Protocol (ISP):

1. Updated the study timeline (section 7). Enrollment for this study will begin in October 2017.

2. Revised the study personnel roles to include the responsibilities of the Department of pediatric study Clinical Research Assistant (CRA) (section 22) who has been added to the protocol as of September 2017.

3. We also added a study flyer to use for recruitment purposes. A copy of the flyer has been included in section 7. Also, we have made the decision to compensate subjects with $50 cash after each study visit. The initial ISP stated that bookstore gift cards would be given as compensation after each study visit. We've since learned that cash is more flexible for parents and subjects and the preferred method of payment (section 23).

The initial ISP described the use of a telephone script for follow up phone calls. We have included that script in section 7.

Submission of study revision 3 on 12/28/2017 which is summarized as follows:

Changes to the ISP:

We requested the IRB to allow us to change the duration of the Run-in phase of the study (Visit 3) from 4 weeks, to 2-4 weeks. This change is requested to decrease the amount of time that subjects must wait prior to study randomization. We do not sense that lessening the run-in phase will increase the risk to subjects enrolled since the Treat-to-target regimen that takes place during the run-in phase of this study (Visit 3) is

based on the basal-bolus regimen that is considered standard treatment for children and adults with T1D.

Revisions were made to Table 1 (Pg7) to clarify the mixed meal tolerance test (MMTT) visits, by removing “dispense insulin and supplies” since insulin is not dispensed as part of this study and other minor editorial clarifications.

Changed the timing/scheduling of the MMTT. The Clinical Research Center (CRC) does not open until 8:30am so we had to change the testing time from 7:00am-10:00am to 8:30am-10:00am.

We wanted the nutrition visit to be consistent with the language on page 9 of the ISP and to simply state that the first nutrition visit will take place within 2 months of enrollment which may not necessarily be during the visit 3 study visit.

On 6/14/2018, the ISP was revised as follows: Inclusion and exclusion criteria: In the ISP (Page 4, under inclusion criteria), the last point originally read "9. Fasting C-peptide level of >0.1 nmol/L (0.3 ng/mL)[19]".
This sentence was incomplete, and the actual point should have read:
"9. Fasting C-peptide level of >0.1 nmol/L (0.3 ng/mL)[19] or 2-hour post-meal stimulated C-peptide level of 0.2 nmol/L (≥0.6 ng/mL)." These are the criteria which we listed elsewhere in the study documents, for example, the document titled "Human Subjects Protection". So, we are correcting this point so all the study documents will contain the same criteria.

To be compliant with the consent witness procedures, we are clarifying in the ISP that witness signature is only required if study participants are not English speaking. This language appears on page 23 of the ISP.

On 7/21/2018, we requested the IRB to allow us to modify our protocol to allow for an immediate processing of the C-peptide sample collected during visits 4-8, as is already being done for visit 2. Though we had originally planned to run these assays at the end of the study, we have learnt that C-peptide does degrade in the first year of storage even in the best of conditions. We are making this modification to ensure the validity of our C-peptide assay results. We will also want to begin to run the C-peptide after each visit going forward. We have modified the ISP which is the only applicable document for this change of timing of assay processing for C-peptide.

On, 11/30/2018, the ISP was revised: The ISP states that the first morning void is required for VDBP testing during study visits. In practice many patients do not bring in their first morning void and a spot urine is collected instead. Though the first morning void is preferable, the spot urine is an acceptable secondary option if the morning void is not supplied. To correct this the ISP was modified to state that a morning void is preferable, but not required, for vitamin D binding protein (VDBP) testing, and that a spot urine will be accepted if the morning void is not supplied.

Changes to initial administration of study drug and safety labs. The time to start the initial dose of study drug was adjusted to allow for communication of the result of serum 25-hydroxyvitamin D [25(OH)D] to the family. Thus, the first dose is to be taken after 25(OH)D levels drawn at visit 4 are received and verified to be < 70ng/mL. Additionally, visit 4b (a now redundant safety visit) was removed.

Improved clarity of payment scheme, and the acceptance of urine sample during visit in situations where the first morning urine void was not available

On 12/4/2018, Exclusion criteria #4 was included as we did not want to knowingly recruit any subjects with existing 25(OH)D levels that were greater than 70 ng/mL. Please note that this was not a requirement for screening visits, but a review criterion from existing records. In response to the audit on 11/28/2018, as well as given the fact that the DSMB report from 11/27/2018 did not show any evidence of vitamin D toxicity associated with this study, we agree with the auditor to remove this component of the exclusion criteria as we have not encountered any cases of elevated 25(OH)D in study subjects. To correct this, we have decided to remove this exclusion criteria in the protocol. Additionally, we will use an inclusion/exclusion criteria checklist to prevent this type of oversight in any subjects recruited in the future.

On 12/18/2018, There are 2 reasons for this modification, and both are listed below:
1. We are continuing our study as originally planned without the inclusion of patients with type 1b diabetes. This was to ensure that all subjects had positive diabetes-associated antibodies in serum before they are enrolled in the trial.
2. We have reinstated the following exclusion criteria: "Subjects with 25(OH)D levels of >70 ng/mL, as this may lead to vitamin D toxicity in the study subjects". Screening for 25(OH)D levels of >70 ng/mL was requested by the IRB to be done prior to subjects receiving their initial dose of the study drug (vitamin D or placebo). This safety lab will now be performed at visit 4. The ISP has been updated to reflect that baseline 25(OH)D will be obtained at Visit 4 and initial administration of study drug will occur after a review of the safety lab tests to ensure that the subjects do not have a 25OHD of >70 ng/mL. As a result of this request from the IRB, visit 4b now occurs at Visit 4 (randomization) and visit 4a will now occur 4-6 weeks after randomization.

On, 2/19/2019, We are making changes to the ISP to include the use of EMLA cream (for needle-phobia) and other similar devices to reduce discomfort during blood draws.

Additionally, we created a document that displays the subjects’ number of visits, research procedures and time commitment and compensation at each visit.

Though the ISP mentions the drawing of safety labs these are not specifically referred to as visits 4a and 4b. Additionally, the compensation scheme for the safety visits is mentioned, but not clear, as it differs from the compensation scheme of the core study

visits: V1, V2, V3, V4-V9. The compensation scheme for these visits is explicitly stated in the ISP. To correct this, we will add visits 4a and 4b to the ISP and clarify that the compensation is only a parking voucher, rather than $50.00 and a parking voucher. The ISP now includes changes in visit 4 to make it easier for auditors to understand.

On 4/25/2019, the study protocol was revised because though the original protocol stated the patients would be weighed in their undergarments and with no shoes, but we felt that it wasn't required that a patient needed to undergo this for the physical and history examination segment of their visit. All patients to date have been weighed in loose, lightweight clothing with no shoes. A protocol modification was then submitted to change the ISP to state subjects would be weighed in loose, lightweight clothing.

On 6/10/2019 the study protocol was revised. This was because of a handful of subjects who dropped out of the study after enrollment but before randomization. Therefore, to meet our original goal of 40 randomized patients, we increased the total number of subjects enrolled from 40 to 48 (an increase of 8 subjects). We have selected 48 to account for both the subjects that have already dropped out prior to randomization (n=6), and any that may drop out in the future.

Additionally, we added lipid panel to the baseline labs drawn during visits 4, 5, 6, 7, and 8. We wanted the results of the lipid profile to enable us to obtain data on non-glycemic metabolic parameters which are also important for the assessment of cardiovascular health of patients with type 1 diabetes.

On, 9/3/2019 the ISP was revised to clarify that the issue of urine calcium and creatinine. The current protocol states that urine Ca will be collected at visits 4, 4a, 5, 6, 7, and 8, but did not mention creatinine. As the sample that is collected to run the urine calcium assay also requires an assay for urine creatinine for validation, we have been running into issues with our research billing department as they are having difficulty billing for the urine Ca/Cr ratio. Currently the assay for urine creatinine is designated as Research Study (RS) on the billing grid for study visits 4, 5, 6, 7, and 8, however it is undesignated for V4a. To correct this, the modification is changing all mentions of "urine Ca" in the protocol to "urine Ca and Creatinine". These changes would clarify that it is both urine calcium and creatinine that are to be collected at visits 4, 4a, 5, 6, 7, and 8.

In addition to the above change, we have also decided to extend the window of safety visit 4a. Currently in the protocol we are limited to a 2-week window as it states that visit 4a will occur 4 - 6 weeks after visit 4. As we have had difficulties getting subjects to come in for their safety lab draws during this window, we would like to extend this window. The protocol was changed to state that the safety draw will occur 4 - 10 weeks after randomization.

On 1/6/2020, ISP was revised to enable us collect continuous glucose monitoring (CGM) data. In addition to the data collected from the Precision Xtra Blood Glucose

Monitoring System (Abbott Diabetes Care, Alameda, CA, USA) and the subject’s insulin pump, continuous glucose monitoring (CGM) data will also be collected for those subjects that are using CGM. By collecting the CGM data, the subject's blood sugar levels can be monitored, and evaluated throughout the study. It is crucial for subjects to have healthy blood sugar levels throughout the day and night, so by collecting CGM data, the subjects will be evaluated and treated for blood sugar levels that are too high or too low.

On 10/8/2020, ISP was revised to enable us to expand the cytokine panel to include 3 additional cytokines to enable the investigators to compare the levels of pro-inflammatory and anti-inflammatory cytokines
