# Supplementary material for: Effect of Ergocalciferol on β-Cell Function in New-Onset Type 1 Diabetes: A Secondary Analysis of a Randomized Clinical Trial
Source: JAMA Netw Open. 2024 Mar 5;7(3):e241155. doi: 10.1001/jamanetworkopen.2024.1155 (PMC10915693; doi:10.1001/jamanetworkopen.2024.1155)
Supplement: Supplement 3. — eFigure. CONSORT Flow Diagram [file jamanetwopen-e241155-s003.pdf]

Supplementary Online Content

Nwosu BU, Parajuli S, Sharma RB, Lee AF. Effect of ergocalciferol on β-cell function in new-onset type 1 diabetes. *JAMA Netw Open*. 2024;7(3):e241155. doi:10.1001/jamanetworkopen.2024.1155

eFigure. CONSORT Flow Diagram

This supplementary material has been provided by the authors to give readers additional information about their work.

© 2024 Nwosu BU et al. *JAMA Network Open.*

CONSORT
TRANSPARENT REPORTING of TRIALS

CONSORT 2010 Flow Diagram

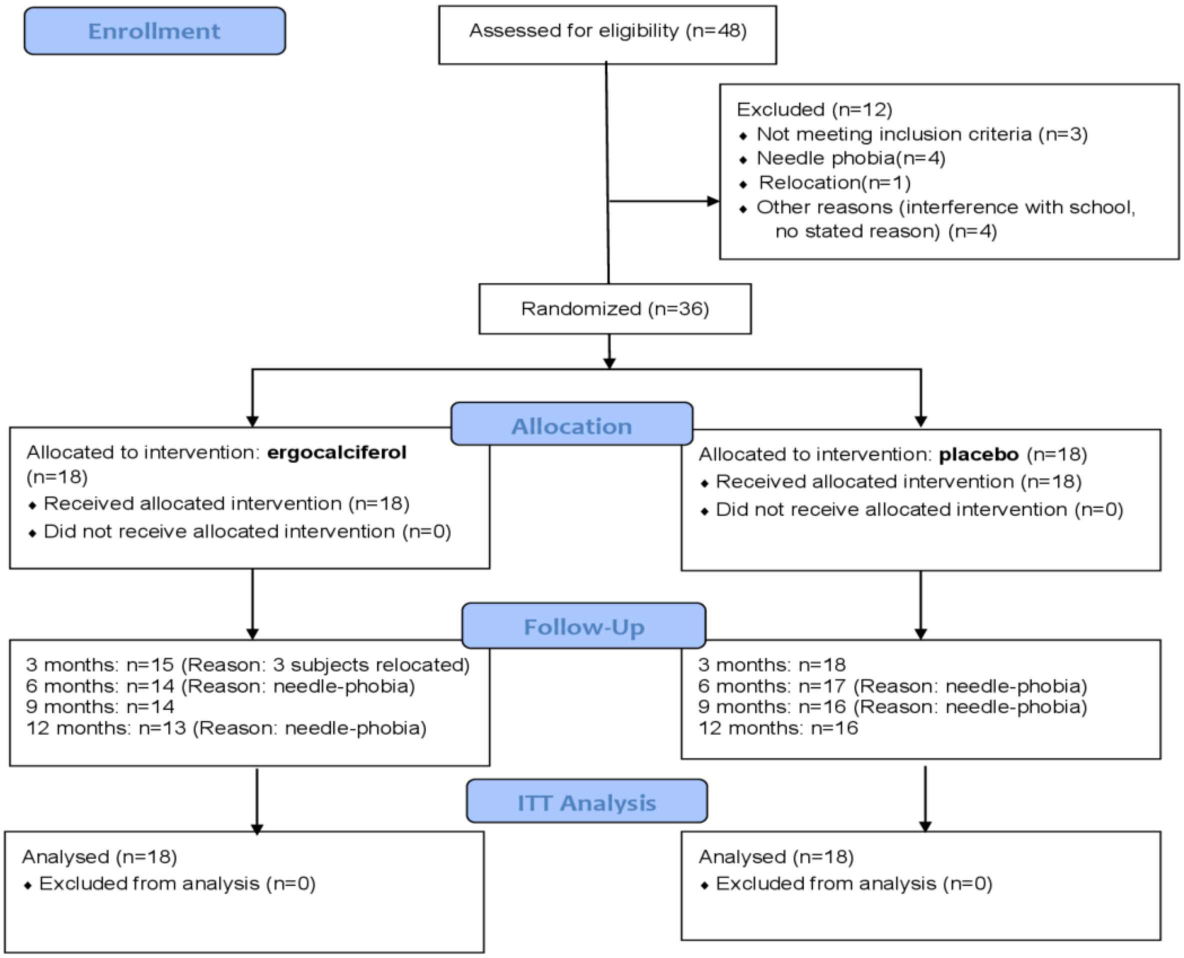

© 2024 Nwosu BU et al. *JAMA Network Open.*
