# Supplementary material for: Effect of Ergocalciferol on β-Cell Function in New-Onset Type 1 Diabetes: A Secondary Analysis of a Randomized Clinical Trial
Source: JAMA Netw Open. 2024 Mar 5;7(3):e241155. doi: 10.1001/jamanetworkopen.2024.1155 (PMC10915693; doi:10.1001/jamanetworkopen.2024.1155)
Supplement: Supplement 4. — Data Sharing Statement [file jamanetwopen-e241155-s004.pdf]

Data Sharing Statement

Nwosu. Effect of Ergocalciferol on β-Cell Function in New-Onset Type 1 Diabetes. *JAMA Netw Open*. Published March 05, 2024. doi:10.1001/jamanetworkopen.2024.1155

Data
Data available: Yes
Data types: Other (please specify)
Additional Information: Study protocol
How to access data: Nwosu B.U.; Parajuli. S, Jasmin, G., Fleshman, J., Sharma RB, Alonso LC, Lee AF, Barton BA 2021 Investigational Study Protocol: https://escholarship.umassmed.edu/datasets/3/.
When available: With publication

Supporting Documents
Document types: None

Additional Information
Who can access the data: Researchers whose proposed use of the data has been approved.
Types of analyses: Deidentified data analyses
Mechanisms of data availability: Data will be made available with a signed data access agreement.
Any additional restrictions: None
